# XLA-MTS: a distinct clinical genetic entity characterized by immunodeficiency and neurodevelopmental delay

**DOI:** 10.1186/s13023-026-04447-7

**Published:** 2026-06-25

**Authors:** Ignacio Ventura, Francisco Revert-Ros, Fernando Revert, Juan Carlos Marín-Paya, Natalia Ruíz-Pérez, Jesús Ángel Prieto-Ruiz, José Miguel Hernández-Andreu

**Affiliations:** Grupo de Medicina Molecular y Mitocondrial, Facultad de Medicina y Ciencias de la Salud, Universidad Católica de Valencia San Vicente Mártir, c/Quevedo 2, Valencia, 46001 Spain

**Keywords:** Mohr–Tranebjærg, *TIMM8A*, *BTK*, XLA-MTS, Deafness-dystonia syndrome, Contiguous deletions, ORPHA99707

## Abstract

**Background:**

Mohr–Tranebjærg syndrome (MTS) is a rare X-linked recessive neurodegenerative disorder, typically presenting with progressive sensorineural hearing loss in early childhood, followed by neurological deterioration, dystonia, ataxia, and visual impairment.

**Objective:**

This study aimed to conduct a comprehensive clinical, genetic, and phenotypic review of MTS, with particular emphasis on mutations in the *TIMM8A* gene and contiguous deletions involving Bruton tyrosine kinase (*BTK*), which give rise to an extended phenotype known as XLA-MTS.

**Methods:**

A systematic literature review was performed between February 1995 and May 2025 using PubMed, Embase, and Scopus databases. Clinical and genetic data from 75 individual patients with molecularly confirmed MTS or XLA-MTS were extracted and stratified into two groups: classical MTS (*TIMM8A* mutations only, *n* = 51) and XLA-MTS (contiguous deletions of *TIMM8A* and *BTK*, *n* = 24). Key clinical features were compared using Fisher’s exact test and Student’s t-test.

**Results:**

Immunodeficiency with agammaglobulinemia (XLA) was present in 100% of XLA-MTS cases (24/24) versus 0% of classical MTS cases (0/51). Delayed language development (DLD) was significantly more frequent in XLA-MTS (52,4%, 13/24) than in classical MTS (19.6%, 10/51) *(p* = 0.004). In contrast, progressive cortical blindness (PCB) was markedly more common in classical MTS (58,8%, 30/51) than in XLA-MTS (12,3%, 3/24) (*p* < 0.001).

**Conclusions:**

Contiguous deletions encompassing *TIMM8A* and *BTK* define a distinct clinical-genetic entity—XLA-MTS—characterized by the triad of early-onset deafness, neurodevelopmental delay, and immunodeficiency. These findings underscore the critical need for expanded genetic testing beyond *TIMM8A* alone to ensure accurate diagnosis, prognostic stratification, and appropriate clinical management, particularly in patients with syndromic deafness and developmental delay.

**Supplementary Information:**

The online version contains supplementary material available at https://doi.org/10.1186/s13023-026-04447-7.

## Introduction

Mohr–Tranebjærg syndrome (MTS) is a rare X-linked recessive disorder characterized by early-onset progressive sensorineural hearing loss during childhood, followed by neurodegeneration that broadens the phenotypic spectrum to include ataxia, dystonia, and visual impairment [44]. Also referred to as deafness-dystonia syndrome (DDS) or deafness-dystonia-optic neuropathy (DDON) syndrome [47]. MTS is caused by mutations in the *TIMM8A* gene, which encodes the mitochondrial inner membrane translocase protein Timm8a [36].

Through linkage analysis, the causative locus was mapped to Xq22 and subsequently associated with a pathogenic variant in *TIMM8A* [18]. *TIMM8A* is a small gene composed of two exons encoding a 97-amino acid protein that functions as a mitochondrial inner membrane translocase. The Timm8a protein, formerly known as DDP1, shares both intracellular localization and functional similarity with the *Saccharomyces cerevisiae* gene *YJR135w-A*, referred to as Tim8p. Early functional studies using this yeast homolog provided critical evidence that MTS is a mitochondrial disorder resulting from a defective protein import system [22].

A range of *TIMM8A* variants has been described, including missense, nonsense, splice-site variants, small insertions/deletions, as well as partial and complete gene deletions. Notably, contiguous gene deletions involving *TIMM8A* account for a significant proportion of the reported variants. Yeast studies have demonstrated that the *TIMM8A* homolog, Tim8, localises to the mitochondrial intermembrane space, where it forms a 70 kDa hetero-oligomeric complex with Tim13 [10].

The Tim8a–Tim13 complex plays a pivotal role in stabilising the precursor form of Tim23, a key component of the mitochondrial inner membrane translocase [9, 2]. Truncating mutations or the C66W missense variant in TIMM8A disrupt the assembly of this complex [17], thereby impairing hTim23 biogenesis in affected tissues [36]. Although the molecular mechanisms underlying Mohr–Tranebjaerg syndrome (MTS) remain incompletely understood, altered Tim23 function is thought to contribute directly to disease pathogenesis [34]. In parallel, dysfunction of mitochondrial respiratory chain complexes—particularly Complex II—has also been implicated as part of the pathogenic cascade associated with this disorder [13].

Beyond its role in protein translocation, the Tim8a–Tim13 complex is involved in the import of specific mitochondrial carrier proteins. In mammalian mitochondria, its substrates include aspartate–glutamate carriers such as citrin and aralar1, key components of the NADH-dependent malate–aspartate shuttle [10]. The co-expression of aralar1, TIMM8A and TIMM13 in cerebellar neurons, together with NADH depletion and defective hTim23 biogenesis, provides a plausible molecular framework for the selective neuronal vulnerability observed in MTS [37].

Furthermore, emerging evidence suggests that hTim8a has additional functions in mitochondrial homeostasis that extend beyond protein import. Although its precise role in neurons and its direct link to neurodegeneration remain to be fully elucidated, hTim8a has been shown to be essential for the assembly of mitochondrial Complex IV through interactions with assembly factors such as the copper chaperone COX17 [20]. Loss of hTim8a may therefore promote oxidative stress and disrupt apoptotic signalling pathways, ultimately contributing to the neurodegenerative phenotype characteristic of MTS [20].

Notably, we recently described a novel hemizygous frameshift variant in TIMM8A (c.98_101dupAGCA; p.Leu35fs) in a male adolescent presenting with progressive dystonia and basal ganglia iron deposition on MRI, but without sensorineural hearing loss—a striking deviation from the canonical MTS phenotype. This case not only expands the mutational and clinical spectrum of TIMM8A-related disorders but also raises the possibility of a previously underappreciated role for TIMM8A in cerebral iron homeostasis [49].

Another important gene to highlight is BTK, which is commonly included in contiguous gene deletions located less than 1 kb downstream of TIMM8A [3, 38]. Larger deletions involving additional genes in the Xq22.1 region have also been reported, underscoring the complex genomic architecture of this locus.Pathogenic variants in *BTK* cause immunodeficiency due to agammaglobulinemia XLA [34]. XLA is characterised by early onset of recurrent bacterial infections and a marked reduction in circulating B cells [34]. Clinically, the syndrome known as XLA-MTS results from contiguous gene deletions involving both *TIMM8A* and *BTK*, presenting a phenotypic spectrum encompassing features of both MTS and XLA [34].

In hemizygous males for pathogenic variants in *TIMM8A*, the majority develop the typical MTS phenotype with variability in severity and age of onset; we have not found cases of pathogenic mutation carriers who do not manifest the disease at some point in their lives. In heterozygous females, expression is generally mild or absent, modulated by skewed X-inactivation [31, 32]. For *BTK*, loss of function in males confers virtually complete penetrance of XLA, whereas female carriers are asymptomatic in most cases. However, skewed X chromosome inactivation may lead to immunodeficiency in female heterozygous carriers of pathogenic mutations in *BTK* [14].

Main objective: to analyse the genetic, molecular, and clinical foundations of MTS and its XLA-MTS variant, with special emphasis on contiguous gene deletions involving *TIMM8A* and *BTK*, through a critical review of the scientific literature and reported clinical cases. To characterise the clinical phenotype of MTS, describing its primary neurological, auditory, and visual manifestations, as well as the variability associated with underlying genetic alterations. To analyse the pathogenic variants reported in the *TIMM8A* gene, including a comparison between point mutations and larger deletions encompassing contiguous genes. To evaluate the clinical implications of deletions involving *TIMM8A* and neighboring genes such as *BTK*, especially regarding the development of immunodeficiency (XLA-MTS), and to determine how these deletions contribute to a more complex phenotypic spectrum.

## Materials and methods

### Systematic literature review and case selection

We conducted a systematic literature review in accordance with established methodological standards for case-based reviews of rare diseases following the PRISMA 2020 guidelines [29]. This systematic review was not prospectively registered.

Electronic searches were performed in PubMed, Embase, and Scopus from February 1995 to May 2025. The following search terms were used, alone or in combination: “Mohr–Tranebjærg syndrome,” “*TIMM8A*,” “DDP1,” “deafness-dystonia syndrome,” “X-linked agammaglobulinemia,” “contiguous gene deletion,” and “clinical case.” A total of 47 peer-reviewed publications reporting 75 individual patients with genetically confirmed Mohr–Tranebjærg syndrome (MTS) or its variant XLA-MTS were included in the final analysis.

**Inclusion criteria were:**

- Full-text availability in English or Spanish;
- Availability of individual-level clinical and genetic data;
- Molecular confirmation of pathogenic variants in *TIMM8A* or in zinc *TIMM8A* and contiguous genes (e.g., *BTK*).

**Exclusion criteria comprised:**

- Studies lacking sufficient phenotypic detail (e.g., absence of age at onset, symptom description, or genotype);
- Articles focused exclusively on molecular or cellular mechanisms without patient-level data;
- Duplicate reports of the same patient across multiple publications.

References listed in Table S2 were excluded from the clinical case review because they did not provide individual-level patient data; however, some of these sources are cited in the Introduction and Discussion to illustrate molecular mechanisms or experimental findings relevant to Mohr–Tranebjærg syndrome.

From each eligible case, we extracted the following variables: core clinical manifestations (sensorineural hearing loss, dystonia, ataxia, visual impairment, immunodeficiency, neurodevelopmental delay), genetic alteration type (point mutation, frameshift, splice-site, intragenic deletion, or contiguous deletion), affected gene(s), and diagnostic methods used (e.g., Sanger sequencing, qPCR, array CGH, NGS). Data were systematically compiled into a standardized matrix to enable genotype–phenotype correlation (Table S1).

Cases were included only when both molecular confirmation of *TIMM8A* involvement and compatible clinical manifestations of Mohr–Tranebjærg syndrome were reported. Clinical features considered compatible with MTS included progressive sensorineural hearing loss, dystonia, ataxia, visual impairment, neurodevelopmental delay, and related neurological manifestations, as described by the original authors. Applying ACMG-AMP 2015 criteria, most variants in the cohort were classified as pathogenic due to their predicted loss-of-function effect (PVS1) in *TIMM8A*, a gene with a well-established LOF disease mechanism. In addition, all variants met the PS4 criterion, as they were consistently observed in affected individuals with Mohr–Tranebjaerg syndrome (MTS). The phenotypic consistency across patients (PP4) further supported this classification. Intronic variants were classified as likely pathogenic due to suspected splicing effects, although lacking functional validation. In contrast, missense variants remained variants of uncertain significance due to insufficient evidence of pathogenic mechanisms.

Genetic testing methodologies reported across the included studies evolved over time, ranging from targeted Sanger sequencing and quantitative PCR (qPCR) to chromosomal microarray analysis and next-generation sequencing (NGS). Recent studies increasingly employed combined approaches (e.g., long-range PCR followed by NGS) to precisely delineate breakpoints in large structural variants, particularly contiguous deletions involving *TIMM8A* and neighboring genes such as *BTK*.

### Statistical analysis

To evaluate phenotypic differences between genetic subtypes, we stratified the cohort into two mutually exclusive groups:

- Classical MTS (*n* = 51): patients with pathogenic variants restricted to *TIMM8A*.
- XLA-MTS (*n* = 24): patients with contiguous deletions encompassing both *TIMM8A* and *BTK*.

The key clinical features were analyzed as binary outcomes (present/absent). Frequencies and percentages were calculated for each feature within each subgroup. Between-group comparisons were performed using Fisher’s exact test, given the small sample size of the XLA-MTS group. For continuous variables (e.g., age at onset of sensorineural hearing loss), we calculated means and standard deviations (SD) and compared groups using the two-tailed Student’s t-test with unequal variances.

All statistical analyses were performed using IBM SPSS Statistics® (Version 31). Categorical variables were analyzed using Fisher’s exact test, while continuous variables were compared using Student’s t-test. A *p*-value < 0.05 was considered statitically significant. Microsoft Excel (*Microsoft 365*) was utilized for data management and chart preparation.

## Results

**Fig. 1:**
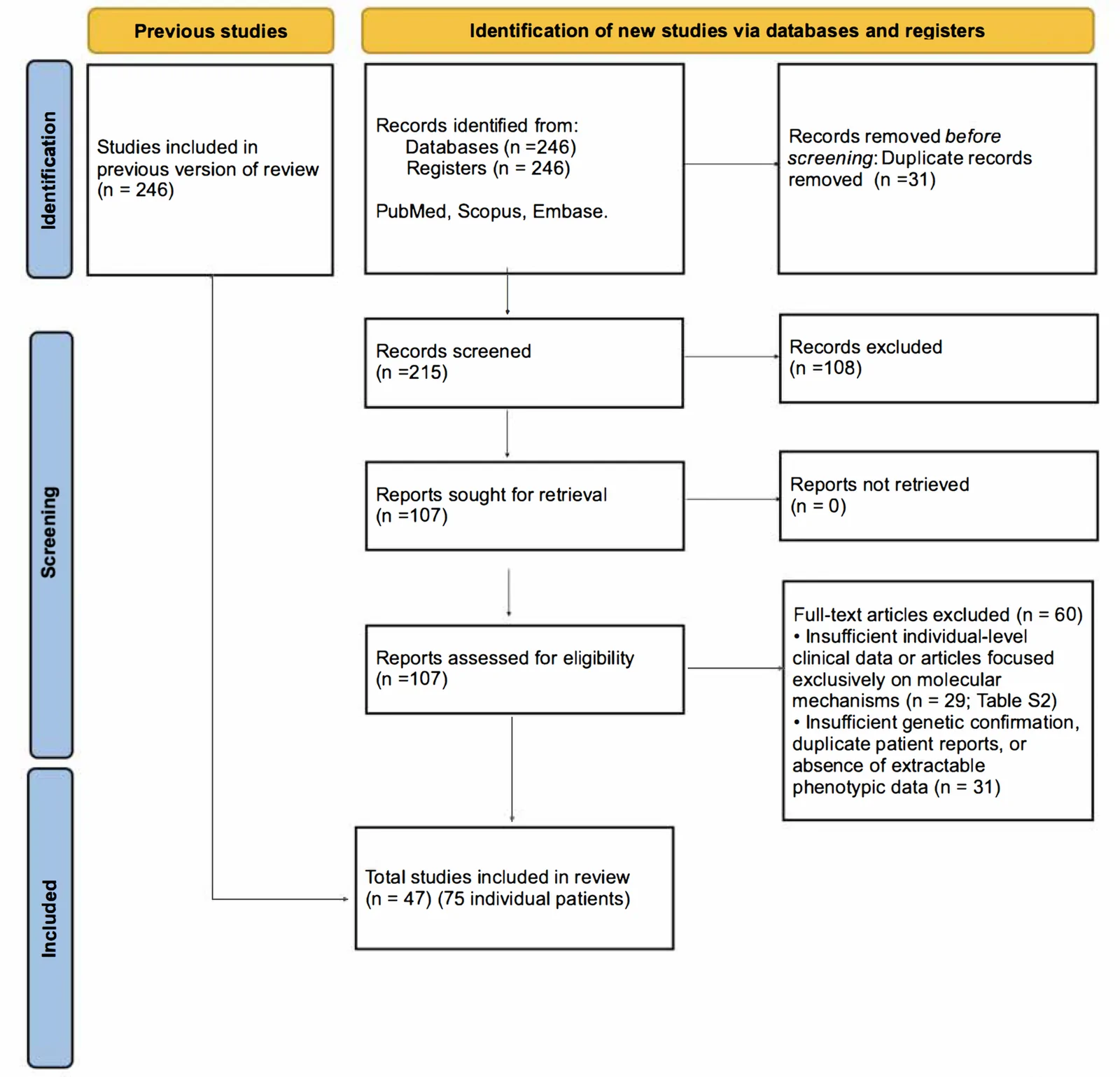
PRISMA flow diagram of study selection: a total of 246 records were initially identified through database searches (PubMed, Embase, Scopus). After removing 31 duplicates and studies that did not meet the inclusion criteria, 47 articles representing 75 patients with MTS or MTS-XLA were ultimately selected for analysis

In our database search we found a total of 246 records, 31 of which were duplicates that were therefore discarded. Of the remaining 215 records, 108 were excluded after titles and abstracts were checked. Of remaining 107 articles, 60 were excluded for several reasons: 29 lacked individual patient-level clinical data or focused exclusively on molecular mechanisms (see Table S2), and 31 were excluded for lacking genetic confirmation, for duplicate reporting of the same patient, or for absence of extractable phenotypic data. Finally, 47 studies met inclusion criteria and were included in the qualitative and quantitative synthesis, comprising a total of 75 individual patients suffering from either MTS or MTS-XLA (Fig. 1).

**Fig. 2:**
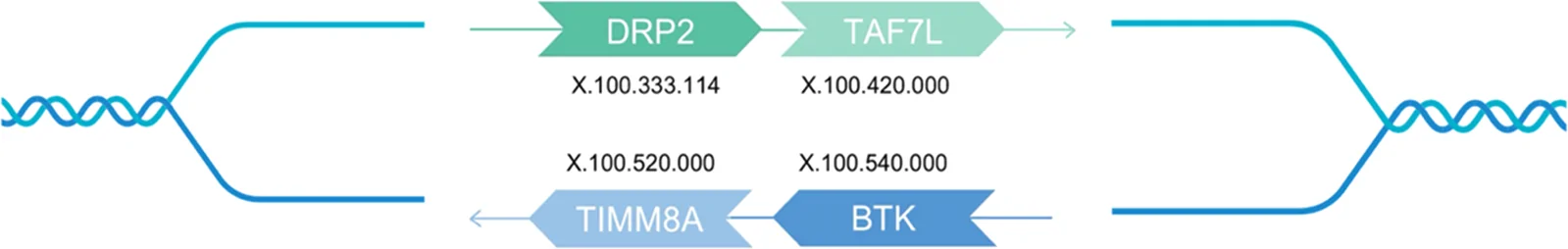
Genomic architecture of the Xq22.1 locus harboring *TIMM8A* and *BTK* genes: schematic representation of key genes within the Xq22.1 region, depicted in their natural orientation on the DNA strand. DRP2 and TAF7L are transcribed in the 5’→3’ direction, while TIMM8A and BTK are oriented 3’→5’. This genomic organization underlies the pathogenic mechanism of contiguous gene deletions causing XLA-MTS

The genomic proximity of *BTK* and *TIMM8A* within the Xq22.1 locus has been well documented in clinical and molecular databases such as MedlinePlus (https://medlineplus.gov) and OMIM (https://omim.org), which provide background information on gene function and associated disorders. This structural arrangement underlies the pathogenic mechanism of contiguous deletions described in Arai et al. [3] and other studies. Figure 2 details the relevant genes in this region according to their natural orientation on the DNA strand: *DRP2* and *TAF7L* oriented 5’→3’, while *TIMM8A* and *BTK* are positioned 3’→5’. This representation is essential for understanding the contiguous deletions affecting multiple genes in this area. Although *DRP2* (Dystrophin-Related Protein 2) is primarily associated with peripheral nerve myelination and not mitochondrial function, its inclusion in large Xq22.1 deletions—alongside *TIMM8A* and *BTK*—may contribute to the phenotypic complexity observed in some XLA-MTS cases, underscoring the need for comprehensive genomic analysis in patients with atypical neurodevelopmental or neuromuscular features.

**Fig. 3:**
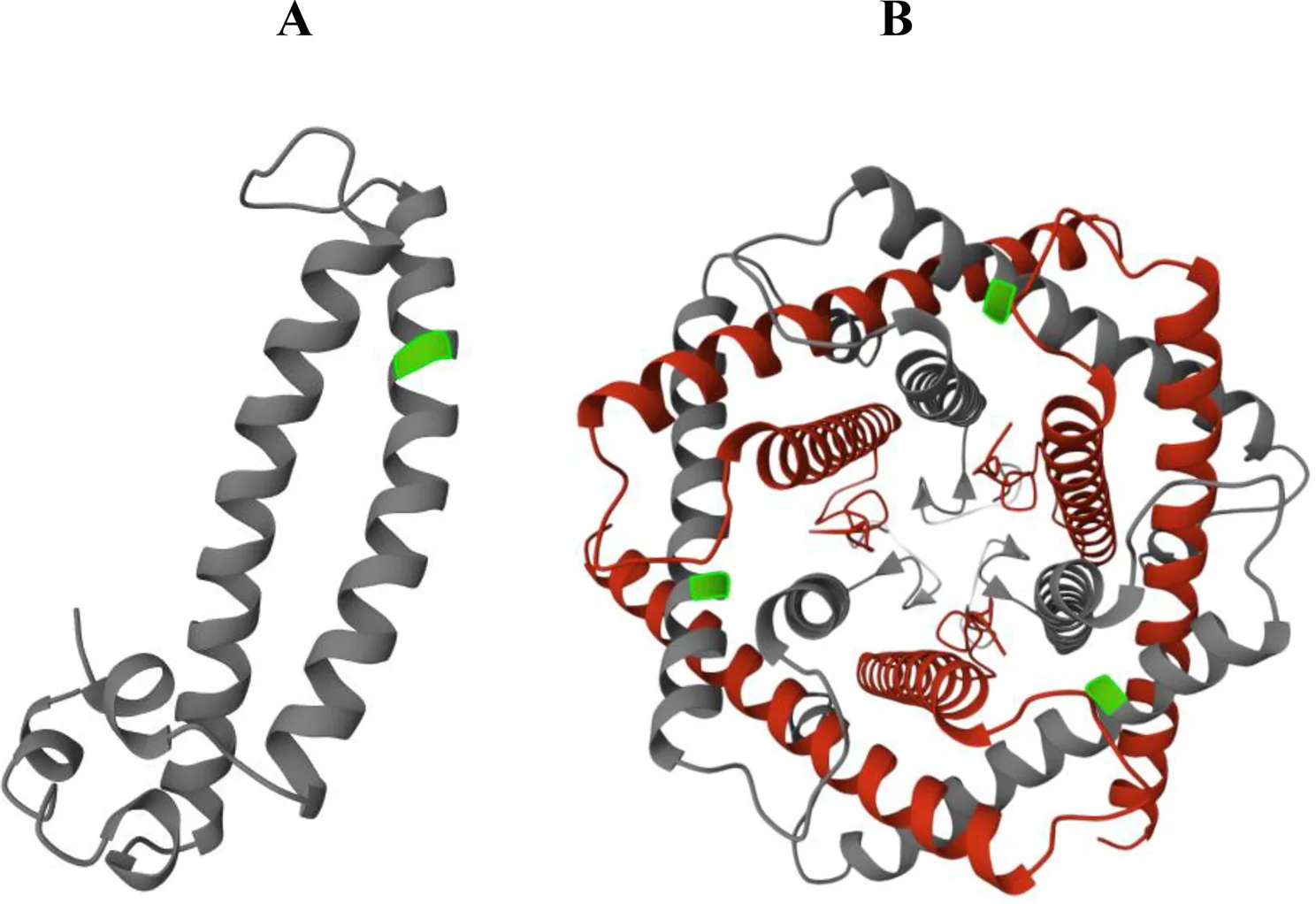
Structural models of TIMM8A protein and TIMM8A-TIMM13 hexamer: In (**A**) 3D structure of the TIMM8A protein highlighting the Cys66 residue. (**B**) 3D structure of the TIMM8A-TIMM13 hexamer. To illustrate the potential structural impact of mutations, three-dimensional models of TIMM8A protein and TIMM8A-TIMM13 hexamer were generated using the Boltz-2 tool and visualized with MolStar application. In both cases, the variant classified as pathogenic by the system was selected. Cys66 residue of TIMM8A is highlighted

To illustrate the potential structural and functional consequences of pathogenic variants, three-dimensional models of the TIMM8A protein (A) and the TIMM8A-TIMM13 hexamer (B) were generated using the Boltz-2 application, accessed via the Tamarind Bio website and visualized using the RCSB PDB’s MolStar application (RCSB PDB − 3D View) (Fig. 3). Panel A highlights the C66 residue in TIMM8A, a critical zinc-binding site frequently mutated in classical MTS (e.g., C66W) [17]. The mutation of this residue impairs the activity of the timm8a/timm13 complex, essential protein import into mitochondria (Fig. 3, panel B).

The clinical characterization of Mohr-Tranebjærg syndrome (MTS) has evolved substantially since its first description by [Mohr and Mageröy [26], who reported X-linked deafness in a large Norwegian family. A pivotal reinvestigation by Tranebjærg et al. [44] redefined the condition as a progressive neurodegenerative disorder characterized by dystonia, cortical blindness, bone fractures, and intellectual disability, and mapped the causative locus to Xq22.

The molecular basis of the disease was established shortly thereafter with the identification of the *DDP* gene (now known as *TIMM8A*) by Jin et al. [18]. Notably, these authors also described the first case of a contiguous gene deletion involving *BTK*, thereby linking MTS with X-linked agammaglobulinemia (XLA). Additional phenotypic variants within this spectrum, including Jensen syndrome, were later confirmed to be allelic forms of MTS following the identification of the E24X nonsense mutation.

Over the past two decades, the description of patients with larger genomic deletions has refined the understanding of the XLA-MTS phenotype. Studies by Richter et al. [35] and Pizzuti et al. [30] reported sporadic cases and demonstrated that complete loss of *TIMM8A* combined with partial loss of *BTK* is associated with earlier onset of cortical blindness and features of muscular dystrophy. Furthermore, analyses of deletions extending up to 196 kb [3, 38] revealed that the involvement of adjacent genes such as *TAF7L* and *DRP2* correlates consistently with severe delayed language development (DLD) and progressive psychomotor impairment.

More recent studies, including those by Ha et al. [15] and Sousa et al. [40], have further delineated the dystonia spectrum in MTS, emphasizing its typically focal or multifocal onset and its progression toward generalized forms.

### Clinical and genetic stratification of Mohr–Tranebjærg syndrome

To further delineate the clinical spectrum of Mohr-Tranebjærg syndrome (MTS), a comparative analysis of phenotypic features was performed between patients with Classical MTS (*n* = 51) and those presenting with the XLA-MTS variant (*n* = 24) (Table 1). Progressive sensorineural deafness (SSP) was identified as the universal hallmark of both cohorts, presenting in 100% of Classical MTS cases and 95.8% of the XLA-MTS cohort, showing no statistically significant difference between the groups (*p* = 0.32). Similarly, no significant variations were observed in the prevalence of involuntary movements and muscle contractures (MICM; 3.9% vs. 4.2%, *p* = 1.000) or cerebellar ataxia (CA; 13.7% vs. 8.3%, *p* = 0.713). However, classical MTS was characterized by a significantly higher prevalence of severe neurological and neuromuscular complications compared to the XLA-MTS variant. Notably, progressive cortical blindness (PCB) was highly prevalent in the classical cohort but sharply reduced in XLA-MTS (58.8% vs. 12.5%, *p* < 0.001). Central nervous system motor abnormalities were also prominently shifted toward Classical MTS, including dystonia (D) (62.8% vs. 25.0%, *p* = 0.003), spasticity (E) (51.0% vs. 8.3%, *p* < 0.001), and ataxia (A) (37.3% vs. 8.3%, *p* = 0.011). Cognitive and neurodegenerative features followed a similar pattern. Progressive mental deterioration (DMP) (60.8% vs. 25.0%, *p* = 0.005) and reduced intelligence (IR) (54.9% vs. 12.5%, *p* = 0.001) were significantly more frequent in classical cases. This correlates with structural neuroimaging and clinical findings, where basal ganglia degeneration (DGB) (27.5% vs. 0.0%, *p* = 0.005) and optic nerve degeneration (DNO) (35.3% vs. 0.0%, *p* = 0.001) were exclusively documented within the classical MTS group. Additionally, bone fractures (FO) and muscular dystrophy (DM) occurred strictly in the classical cohort (23.5% vs. 0.0% for both, *p* = 0.013). Conversely, delayed language development (DLD) was the only non-immunological feature significantly more prevalent in the XLA-MTS group, affecting more than half of the cohort compared to less than a fifth of classical patients (54.2% vs. 19.6%, *p* = 0.004). As expected by definition, immunodeficiency with agammaglobulinemia (XLA) was exclusively restricted to the XLA-MTS cohor.

**Table 1:**
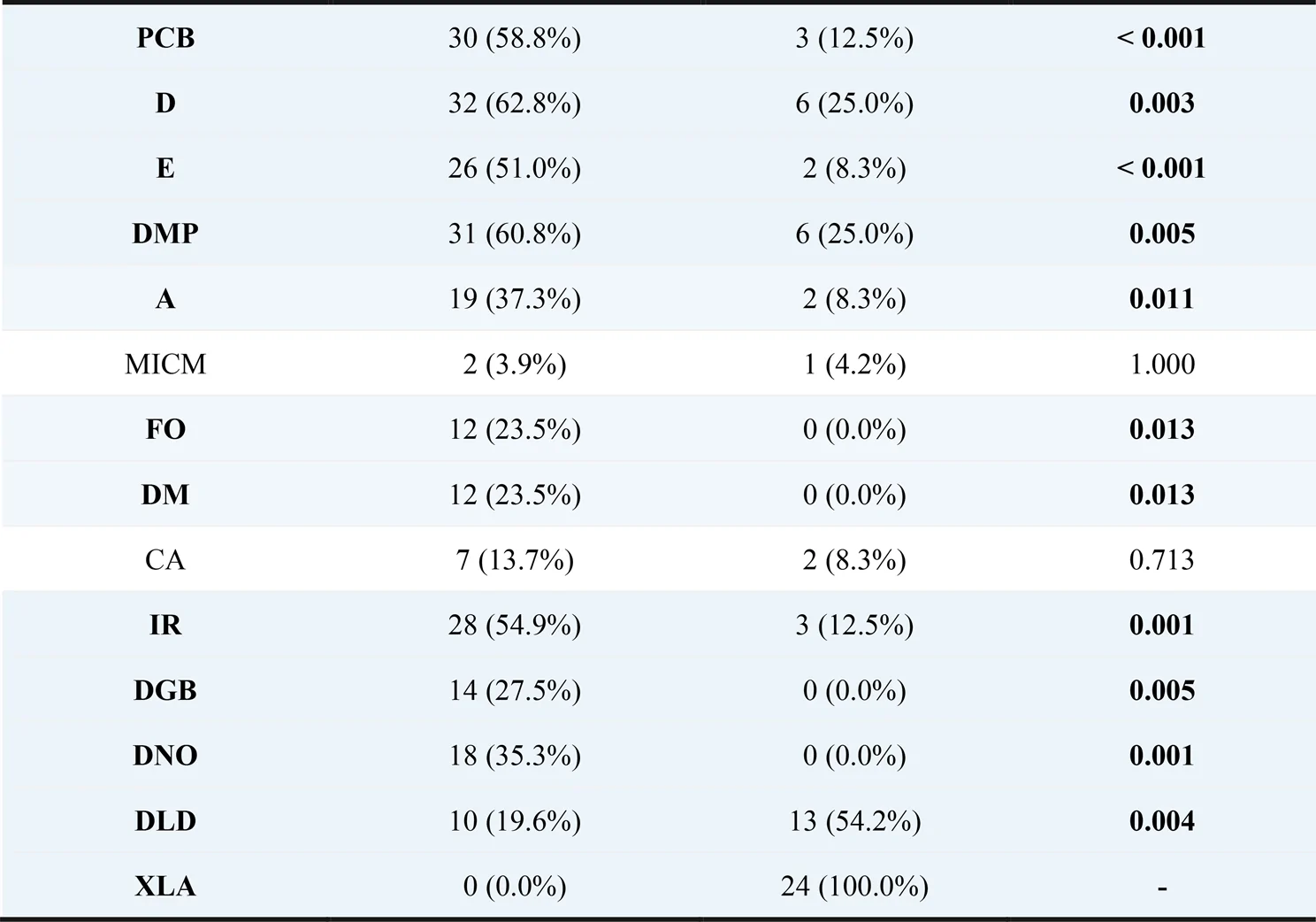
Clinical and Genetic Summary of Reported Cases of MTS and XLA-MTS Included in the Comprehensive Clinical Review: This table summarizes the frequency of the main clinical manifestations observed in patients with classical MTS and XLA-MTS. Comparative analyses were performed using Fisher’s exact test to evaluate differences in symptom prevalence between both groups. Data expressed as absolute frequencies and percentages, n (%). Highlighted rows indicate distinct levels of statistical significance [1–7, 11, 15, 16, 18, 19, 21, 23, 24, 25, 27, 28, 30, 33, 34, 39, 40–46, 48,40–53]

*Legend: SSP = Progressive sensorineural deafness, PCB = Progressive cortical blindness, D = Dystonia, E = Spasticity, DMP = Progressive mental deterioration, A = Ataxia, MICM = Involuntary movements and muscle contractures, FO = Bone fractures, DM = Muscular dystrophy, CA = Cerebellar Ataxia IR = Reduced intelligence, DGB = Basal ganglia degeneration, DNO = Optic nerve degeneration, DLD = Delayed language development, XLA = Immunodeficiency with agammaglobulinemia

Analysis of the 75 compiled cases reveals a clear stratification of clinical phenotypes based on underlying genetic etiology, supporting the existence of distinct clinical-genetic subgroups within the Mohr–Tranebjærg syndrome spectrum.

Classical MTS (*TIMM8A* mutations, *n* = 51) harbored genetic alterations restricted to *TIMM8A*, including 45 cases (88%) with point mutations (missense, nonsense, frameshift, or splice-site) and 3 cases (6%) with partial or complete intragenic deletions. This group exhibits profound clinical heterogeneity. Phenotypes vary widely in both the combination and severity of core features, which include progressive sensorineural hearing loss, dystonia, ataxia, progressive cortical blindness, and cognitive decline. The absence of a unifying clinical pattern beyond early-onset deafness underscores the variable expressivity of *TIMM8A* mutations (Table S1).

In XLA-MTS (Contiguous *TIMM8A*-*BTK* deletions, *n* = 24), conversely, a highly consistent clinical profile was observed in the 24 patients (32%) harboring contiguous deletions. While neurological symptoms like dystonia and ataxia remained present, the definitive hallmark unifying this subgroup was immunodeficiency due to X-linked agammaglobulinemia (XLA), driven by *BTK* haploinsufficiency. Notably, this immunological component was entirely absent in classical MTS cases, establishing a clear phenotypic boundary between the two cohorts.

### Mitochondrial gene mutations: a distinct clinical signature

Cases associated with primary mutations in mitochondrial genes (*MTND4*, *MTND6*, *DNAJC19*; *n* = 5) presented a clinical signature distinct from both classical MTS and XLA-MTS. These patients typically exhibited features consistent with primary mitochondrial dysfunction, including prominent neuromuscular symptoms (e.g., myopathy, exercise intolerance) and metabolic abnormalities, rather than the progressive neurodegenerative course characteristic of *TIMM8A*-related disease. Table 2 summarizes mutations in four distinct genes—PLP1, MT-ND6, MT-ND4, and DNAJC19—and their associated clinical phenotypes in patients with progressive neurological, sensory, and systemic disorders. Each gene plays a critical cellular role in tissues with high energy demands or in the structural integrity of the central nervous system, accounting for the severity and diversity of the observed clinical manifestations.

**Table 2:** Functional roles of genes associated with multisystem neurological and mitochondrial disorders: this table summarizes pathogenic variants in four genes (*PLP1*, *MT-ND6*, *MT-ND4*, and *DNAJC19*) and their associated clinical features, highlighting distinct mechanisms—demyelination and mitochondrial dysfunction—that underlie overlapping multisystem neurological phenotypes

Type of Mutation	Affected Gene	Symptoms Abbreviations	Reference
Possible deletion or point mutation	*PLP1*	SSP, PCB, D, E, DMP, A, FO, DM	[44]
Missense mutation	*MTND6*	D, A, E, IR	[50]
Missense mutation	*MTND4*	PCB, DGB, DNO	[50]
Missense mutation	*MTND6*	SSP, DM, MICM, DLD, A	[12]
Splice site mutation	*DNAJC19*	PCB, A, D, IR, CDT, ADS	[8]

*Legend: SSP = Progressive sensorineural deafness, PCB = Progressive cortical blindness, D = Dystonia, E = Spasticity, DMP = Progressive mental deterioration, A = Ataxia, MICM = Involuntary movements and muscle contractures, FO = Bone fractures, DM = Muscular dystrophy, CA = Cerebellar Ataxia IR = Reduced intelligence, DGB = Basal ganglia degeneration, DNO = Optic nerve degeneration, DLD = Delayed language development, CDT = Early-onset dilated cardiomyopathy, ADS = Sexual development disorders, XLA = Immunodeficiency with agammaglobulinemia

A single case associated with *PLP1* mutation represented a historical outlier. Although previously included within the MTS spectrum because of overlapping neurological manifestations, *PLP1* is now recognized as the cause of Pelizaeus–Merzbacher disease, a distinct X-linked leukodystrophy, and is therefore not considered a true subtype of MTS. *PLP1* mutations were associated with progressive neurological impairment, including deafness, cortical blindness, dystonia, spasticity, ataxia, cognitive decline, fractures, and muscular dystrophy, mainly due to defective CNS myelination.

*MTND6* and *MTND4* mutations affected mitochondrial Complex I, impairing oxidative phosphorylation and ATP production. *MTND6* variants were linked to dystonia, ataxia, spasticity, hearing loss, muscular involvement, and cognitive impairment, whereas *MTND4* mutations were mainly associated with cortical blindness, optic nerve degeneration, and basal ganglia involvement, consistent with LHON-related phenotypes.

*DNAJC19* splice-site mutation caused a severe encephalocardiomyopathy phenotype characterized by cortical blindness, ataxia, dystonia, cognitive impairment, dilated cardiomyopathy, and developmental abnormalities, reflecting altered mitochondrial protein processing and bioenergetics.

Overall, these findings highlight two major pathogenic mechanisms underlying inherited neurological disorders: disrupted central nervous system myelination (*PLP1*) and primary mitochondrial dysfunction (*MTND4*, *MTND6*, *DNAJC19*). Despite different molecular origins, all mutations converge in the impairment of motor, sensory, cognitive, and systemic functions, emphasizing the importance of genotype–phenotype correlations for diagnosis and clinical management.

## Discussion

This study elucidates the clinical spectrum MTS, an X-linked neurodegenerative disorder primarily caused by pathogenic variants in *TIMM8A*. Genomic microdeletions encompassing this locus represent a frequent etiology, characteristically manifesting as early-onset sensorineural hearing loss, dystonia, and progressive neurodegenerative deterioration.The physical proximity of genes within the Xq22.1 chromosomal locus creates a highly specialized architectural landscape where structural alterations translate directly into distinct, multi-systemic clinical features. By systematically mapping specific neurodegenerative, immunological, and neuromuscular phenotypes to individual genetic components within this contiguous region, we can better decipher the pathophysiological determinants that differentiate classical MTS from its contiguous gene deletion variant, XLA-MTS.

### TIMM8A: mitochondrial dysfunction and the neurodegenerative cascade

The pathognomonic neurological presentation of both classical MTS and the XLA-MTS variant is fundamentally driven by the loss of function of *TIMM8A*. This gene encodes the *DDP1*/*TIMM8A* protein, a foundational subunit of the mitochondrial intermembrane space chaperone system. As demonstrated by our structural modeling (Fig. 3A), conserved residues such as C66 are critical for zinc-binding stability; consequently, mutations or microdeletions disrupting this specific domain drastically impair the assembly of the functional *TIMM8A*/*TIMM13* complex, ultimately compromising downstream mitochondrial protein import.The clinical manifestation of this molecular lesion is a progressive, multi-systemic neurodegenerative cascade. Progressive sensorineural deafness (SSP) represents the primary and most penetrant clinical feature of *TIMM8A* haploinsufficiency, occurring across virtually all patients in both studied cohorts. Furthermore, the extensive neurodegeneration characterizing classical MTS—encompassing progressive cortical blindness, dystonia, spasticity, and progressive mental deterioration—reflects the heightened vulnerability of metabolic-intensive neural networks to chronic mitochondrial failure, an observation that closely aligns with previously established pathogenic mechanisms [22, 36].

### BTK: haploinsufficiency and Pathognomonic Immunodeficiency

In contrast to classical presentations, the XLA-MTS phenotype introduces a severe, non-neurological clinical dimension dictated entirely by the structural disruption of the adjacent BTK gene. BTK encodes a cytoplasmic tyrosine kinase indispensable for B-cell lineage development, maturation, and survival signaling (Fig. 3B). Genomic microdeletions spanning into the BTK locus result in functional haploinsufficiency, clinically manifesting as X-linked agammaglobulinemia (XLA).Our comparative analysis confirms that this immunological defect serves as a definitive phenotypic boundary, being present in 100% of the XLA-MTS cohort while remaining entirely absent among classical MTS cases (*p* < 0.001). From a clinical management perspective, the inclusion of BTK within the deleted genomic segment drastically alters patient prognosis and therapeutic paradigms, necessitating lifelong, aggressive immunoglobulin replacement therapy to mitigate the risk of severe, recurrent bacterial infections [3].

### Modifying the phenotypic complexity: flanking loci and developmental discrepancies

A remarkable finding in our comparative analysis is the unexpected discrepancy in neurodegenerative severity between the two cohorts. Patients within the XLA-MTS subgroup displayed significantly lower frequencies of severe central nervous system complications, including cortical blindness *(p* < 0.001), dystonia (*p* = 0.003$), and structural basal ganglia or optic nerve degeneration (*p* = 0.005 and *p* = 0.001, respectively), when compared to classical presentations. Conversely, delayed language development (DLD) was markedly enriched in the XLA-MTS group (54.2% vs. 9.6%, *p* = 0.004).This phenotypic divergence highlights the potential regulatory or modifying impact of neighboring structural variants within the Xq22.1 region, specifically involving *DRP2* and *TAF7L* (Fig. 2). While *DRP2* is primarily recognized for its role in peripheral nerve myelination rather than central mitochondrial dynamics, its disruption within larger, contiguous Xq22.1 deletions may structurally modify neural signaling networks. Large-scale genomic rearrangements involving these flanking loci could induce early neurodevelopmental modifications—such as isolated language delays—or potentially engage in genetic modifier mechanisms that delay or attenuate the chronological onset of *TIMM8A*-mediated neurodegeneration.

This study is subject to limitations inherent to rare diseases. The reliance on case reports introduces publication bias toward more severe phenotypes. Additionally, heterogeneity in molecular diagnostic techniques and pathogenicity criteria limits direct comparison across studies. Finally, the relatively small size of the XLA-MTS subgroup (*n* = 24) reduces statistical power for detecting less frequent associations.

## Conclusions

This study confirms that pathogenic variants in *TIMM8A*—ranging from point mutations to partial or complete deletions—underlie most MTS cases. These variants are associated with a highly heterogeneous clinical spectrum encompassing progressive sensorineural deafness, dystonia, ataxia, visual impairment, and cognitive decline.

Crucially, our analysis identifies contiguous deletions involving both *TIMM8A* and *BTK* as the genetic hallmark of a distinct and clinically cohesive subgroup: XLA-MTS. Unlike classical MTS, this entity is consistently characterized by the triad of early-onset deafness, neurological manifestations (often including developmental delay), and immunodeficiency due to XLA. This immunological component is not merely an occasional comorbidity but a defining feature that fundamentally alters the clinical trajectory and management needs of affected individuals.

These findings underscore that Mohr–Tranebjærg syndrome is not a monolithic disorder. The co-occurrence of neuropathy and immunodeficiency in XLA-MTS represents a unique multisystemic phenotype that demands a different diagnostic and therapeutic approach. Therefore, we strongly advocate for expanding first-line genetic testing beyond *TIMM8A* alone. In patients presenting with syndromic deafness and any neurological or developmental abnormalities, comprehensive analysis—including assessment of the adjacent *BTK* locus via deletion/duplication testing or gene panels—is essential for accurate diagnosis, prognostic stratification, and appropriate genetic counseling.

In summary, recognizing XLA-MTS as a distinct clinical-genetic entity is critical for improving patient outcomes. Future research should focus on establishing standardized diagnostic pathways and exploring targeted interventions for this complex, yet phenotypically recognizable, subgroup. Additionally, the implementation of comprehensive genetic panels and systematic genetic counseling should be prioritized to ensure early detection and informed clinical decision-making for affected families. We also advocate for the development of dedicated clinical and molecular databases for MTS and XLA-MTS to facilitate data sharing, improve genotype–phenotype correlations, and accelerate research toward personalized therapies.

## Electronic supplementary material

Below is the link to the electronic supplementary material.

Supplementary Material 1

## Data Availability

All data supporting the findings of this study are derived from published scientific literature and are fully referenced within the manuscript and its supplementary materials. No new datasets were generated or analyzed in this study. During the preparation of this manuscript, the authors used publicly available databases (PubMed, Embase and Scopus) and standard bibliographic management software for data collection and organization. The authors have reviewed and edited all outputs and taken full responsibility for the content of this publication.

## Abbreviations

MTS: Mohr–Tranebjærg Syndrome
XLA-MTS: X-linked Agammaglobulinemia–Mohr–Tranebjærg Syndrome
*TIMM8A*: Translocase of Inner Mitochondrial Membrane 8A (formerly DDP1)
*BTK*: Bruton Tyrosine Kinase
XLA: X-linked Agammaglobulinemia
DDON: Deafness-Dystonia-Optic Neuropathy Syndrome
SSP: Progressive Sensorineural Deafness
PCB: Progressive Cortical Blindness
D: Dystonia
DMP: Progressive Mental Deterioration
CA: Cerebellar Atrophy
DLD: Delayed Language Development
NGS: Next-Generation Sequencing
