# Supplementary material for: XLA-MTS: a distinct clinical genetic entity characterized by immunodeficiency and neurodevelopmental delay
Source: Orphanet J Rare Dis. 2026 Jun 25;21:323. doi: 10.1186/s13023-026-04447-7 (PMC13587407; doi:10.1186/s13023-026-04447-7)
Supplement: Supplementary file 1 [file 13023_2026_4447_MOESM1_ESM.docx]

Table S1. Individual Genotypic and Phenotypic Profiles of the Study Cohort (N = 75)

*Detailed mapping of clinical symptoms across Part A (XLA-MTS / Contiguous Deletions) and Part B (Classical MTS / TIMM8A mutations)*

**PART A: Patients with XLA-MTS (Contiguous Deletions) [n = 24]**

**ID	Source**

**Sex	Mutation Type**

**Methods Used**

**SSP	PCB D E DMP A MICM	FO DM CA IR DGB	DNO	DLD	XLA**

| **1** | Arai et al. (2011) | M | Contiguous deletion | array CGH / FISH | **+** | - | - | - | - | - | - | - | - - | - | - | - - | **+** |
| --- | --- | --- | --- | --- | --- | --- | --- | --- | --- | --- | --- | --- | --- | --- | --- | --- | --- |
| **2** | Arai et al. (2011) | M | Contiguous deletion | array CGH / FISH | **+** | - | - | - | - | - | - | - | - - | - | - | - - | **+** |
| **3** | Brookes et al. (2008) | M | Contiguous deletion | qPCR | **+** | - | - | - | - | - | - | - | - - | - | - | - **+** | **+** |
| **4** | Jin et al. (1996) | M | Contiguous deletion | Southern blot | **+** | - | **+** | - | **+** | - | - | - | - - | - | - | - - | **+** |
| **5** | Jyonouchi et al. (2008) | M | Contiguous deletion | arrayCGH | **+** | - | - | - | - | - | - | - | - - | - | - | - **+** | **+** |
| **6** | Jyonouchi et al. (2008) | M | Contiguous deletion | arrayCGH | **+** | - | - | - | - | - | - | - | - - | - | - | - **+** | **+** |
| **7** | Pizzuti et al. (2004) | M | Contiguous deletion | PCR / Sequencing | **+** | **+** | **+** | **+** | - | - | - | - | - **+** | **+** | - | - - | **+** |
| **8** | Rendtorff et al. (2022) | M | Contiguous deletion | NGS / Mate-pair | **+** | - | - | - | - | - | - | - | - - | - | - | - - | **+** |
| **9** | Rendtorff et al. (2022) | M | Contiguous deletion | NGS / Mate-pair | **+** | - | - | - | - | - | - | - | - - | - | - | - **+** | **+** |
| **10** | Rendtorff et al. (2022) | M | Contiguous deletion | NGS / Mate-pair | **+** | **+** | **+** | - | - | - | - | - | - - | - | - | - - | **+** |
| **11** | Richter et al. (2001) | M | Contiguous deletion | PCR / Sequencing | **+** | - | - | - | - | - | - | - | - - | - | - | - **+** | **+** |
| **12** | Richter et al. (2001) | M | Contiguous deletion | PCR / Sequencing | **+** | - | - | - | - | - | - | - | - - | - | - | - **+** | **+** |
| **13** | Richter et al. (2001) | M | Contiguous deletion | PCR / Sequencing | **+** | - | - | - | **+** | - | - | - | - **+** | - | - | - **+** | **+** |
| **14** | Šedivá et al. (2007) | M | Contiguous deletion | PCR / Sequencing | **+** | - | - | - | - | - | - | - | - - | - | - | - **+** | **+** |
| **15** | Šedivá et al. (2007) | M | Contiguous deletion | PCR / Sequencing | **+** | - | - | - | - | - | - | - | - - | - | - | - - | **+** |
| **16** | Šedivá et al. (2007) | M | Contiguous deletion | PCR / Sequencing | **+** | - | **+** | **+** | - | - | - | - | - - | - | - | - **+** | **+** |
| **17** | Šedivá et al. (2007) | M | Contiguous deletion | PCR / Sequencing | **+** | - | - | - | **+** | - | - | - | - - | - | - | - **+** | **+** |
| **18** | Šedivá et al. (2007) | M | Contiguous deletion | PCR / Sequencing | **+** | - | - | - | **+** | - | - | - | - - | - | - | - **+** | **+** |
| **19** | Šedivá et al. (2007) | M | Contiguous deletion | Nested PCR | **+** | - | - | - | **+** | - | - | - | - - | - | - | - **+** | **+** |
| **20** | Shaker et al. (2016) | M | Contiguous deletion | Microarray, PCR, Flow Cytometry | **+** | **+** | - | - | - | - | - | - | - - | - | - | - - | **+** |
| **21** | Szaflarska et al (2018) | M | Contiguous deletion | array CGH / MLPA | - | - | - | - | **+** | **+** | **+** | - | - **+** | **+** | - | - **+** | **+** |
| **22** | Yeh et al. (2020) | M | Contiguous deletion | PCR / Flow Cyt. | **+** | - | **+** | - | - | **+** | - | - | - - | - | - | - - | **+** |
| **23** | Yeh et al. (2020) | M | Contiguous deletion | PCR / Flow Cyt. | **+** | - | **+** | - | - | - | - | - | - - | - | - | - - | **+** |
| **24** | Wang et al. (2019) | M | Contiguous deletion | NGS | **+** | - | - | - | - | - | - | - | - - | - | - | - - | **+** |

**PART B: Patients with Classical MTS (TIMM8A mutations) [n = 51]**

| **25** | Aguirre et al. (2006) | M | Frameshift | Spel /Sanger | **+** | - | **+** | - | - | - | - | - | - - | - | - - | **+** | - |
| --- | --- | --- | --- | --- | --- | --- | --- | --- | --- | --- | --- | --- | --- | --- | --- | --- | --- |
| **26** | Aguirre et al. (2006) | M | Frameshift | Spel / Sanger | **+** | - | - | - | **+** | - | - | - | - - | **+** | - - | **+** | - |
| **27** | Aguirre et al. (2008) | M | Intron point mutation | Miniseq / Sanger | **+** | **+** | **+** | - | - | - | - | - | - - | **+** | - **+** | - | - |
| **28** | Binder et al. (2003) | M | Point mutation | Western / Sanger | **+** | **+** | **+** | **+** | - | **+** | - | - | - - | - | - - | - | - |
| **29** | Blesa et al. (2007) | M | Point mutation | CSGE / Sanger | **+** | **+** | **+** | **+** | - | **+** | - | - | - **+** | **+** | - - | **+** | - |

| **ID** | **Source** | **Sex** | **Mutation Type** | **Methods Used** | **SSP** | **PCB** | **D** | **E** | **DMP** | **A** | **MICM** | **FO** | **DM** | **CA** | **IR** | **DGB** | **DNO** | **DLD** | **XLA** |
| --- | --- | --- | --- | --- | --- | --- | --- | --- | --- | --- | --- | --- | --- | --- | --- | --- | --- | --- | --- |
| **30** | Ezquerra et al. (2005) | M | Intron point mutation | RFLP / Sanger | **+** | **+** | **+** | **+** | - | - | - | - | - | - | - | - | - | - | - |
| **31** | Ezquerra et al. (2005) | M | Intron point mutation | RFLP / Sanger | **+** | - | - | - | **+** | - | **+** | - | - | - | - | - | - | - | - |
| **32** | Ha et al. (2012) | M | Exon deletion | Sanger | **+** | **+** | **+** | **+** | **+** | - | - | - | - | **+** | - | **+** | **+** | - | - |
| **33** | Ha et al. (2012) | M | Exon deletion | Sanger | **+** | **+** | **+** | **+** | **+** | - | - | - | - | **+** | - | **+** | **+** | - | - |
| **34** | Hayes et al. (1998) | M | unknown | — | **+** | **+** | **+** | **+** | **+** | - | - | - | - | - | **+** | - | - | - | - |
| **35** | Hayes et al. (1998) | M | unknown | — | **+** | **+** | **+** | **+** | **+** | - | - | - | - | - | - | - | - | - | - |
| **36** | Hayes et al. (1998) | M | unknown | — | **+** | **+** | **+** | - | **+** | - | - | - | - | - | - | - | - | - | - |
| **37** | Jin et al. (1996) | M | Frameshift | Sequencing | **+** | **+** | **+** | **+** | **+** | - | - | **+** | - | - | **+** | - | - | - | - |
| **38** | Jin et al. (1996) | M | Frameshift | Sequencing | **+** | **+** | **+** | **+** | **+** | - | - | **+** | - | - | **+** | - | - | - | - |
| **39** | Jin et al. (1996) | M | Frameshift | Sequencing | **+** | **+** | **+** | **+** | **+** | - | - | **+** | - | - | **+** | - | - | - | - |
| **40** | Jin et al. (1996) | M | Frameshift | Sequencing | **+** | **+** | **+** | **+** | **+** | - | - | **+** | - | - | **+** | - | - | - | - |
| **41** | Jin et al. (1996) | M | Frameshift | Sequencing | **+** | **+** | **+** | **+** | **+** | - | - | **+** | - | - | **+** | - | - | - | - |
| **42** | Kim et al. (2007) | M | Intron point mutation | Sanger | **+** | **+** | **+** | **+** | - | - | **+** | - | - | - | - | - | - | **+** | - |
| **43** | Ma et al. (2024) | M | Point mutation | WES / Sanger | **+** | - | - | - | - | - | - | - | - | - | - | - | - | - | - |
| **44** | Neighbors et al. (2020) | M | Start Loss | qPCR / WB | **+** | - | - | - | - | - | - | - | - | - | - | - | - | **+** | - |
| **45** | Rendtorff et al. (2022) | M | Exon deletion | NGS / Mate-pair | **+** | **+** | - | - | - | **+** | - | - | - | - | - | - | - | - | - |
| **46** | Sousa et al. (2023) | M | Frameshift | WES | **+** | - | **+** | **+** | **+** | - | - | - | - | - | **+** | - | - | **+** | - |
| **47** | Sousa et al. (2023) | M | Frameshift | WES | **+** | - | - | - | - | - | - | - | - | - | **+** | - | - | **+** | - |
| **48** | Sousa et al. (2023) | M | Frameshift | WES | **+** | - | **+** | - | **+** | - | - | - | - | **+** | **+** | - | - | **+** | - |
| **49** | Sousa et al. (2023) | M | Frameshift | WES | **+** | - | - | - | **+** | - | - | - | - | - | **+** | - | - | **+** | - |
| **50** | Sun et al. (2022) | M | Frameshift | NGS | **+** | - | - | - | - | - | - | - | - | - | - | - | - | - | - |
| **51** | Swerdlow et al. (2001) | M | Frameshift | RFLP / Sanger | **+** | - | **+** | - | - | - | - | - | - | - | - | - | - | - | - |
| **52** | Tranebjærg et al. (1995) | M | Frameshift | Linkage / PCR | **+** | - | **+** | **+** | **+** | **+** | - | - | **+** | - | **+** | **+** | **+** | - | - |
| **53** | Tranebjærg et al. (1995) | M | Frameshift | Linkage / PCR | **+** | **+** | **+** | **+** | **+** | **+** | - | - | **+** | - | **+** | **+** | **+** | - | - |
| **54** | Tranebjærg et al. (1995) | M | Frameshift | Linkage / PCR | **+** | **+** | **+** | **+** | **+** | **+** | - | **+** | **+** | - | **+** | **+** | **+** | - | - |
| **55** | Tranebjærg et al. (1995) | M | Frameshift | Linkage / PCR | **+** | **+** | **+** | **+** | **+** | **+** | - | **+** | **+** | - | **+** | **+** | **+** | - | - |
| **56** | Tranebjærg et al. (1995) | M | Frameshift | Linkage / PCR | **+** | **+** | **+** | **+** | **+** | **+** | - | **+** | **+** | - | **+** | **+** | **+** | - | - |
| **57** | Tranebjærg et al. (1995) | M | Frameshift | Linkage / PCR | **+** | **+** | - | **+** | **+** | **+** | - | **+** | **+** | - | **+** | **+** | **+** | - | - |
| **58** | Tranebjærg et al. (1995) | M | Frameshift | Linkage / PCR | **+** | **+** | **+** | **+** | **+** | **+** | - | **+** | **+** | - | **+** | **+** | **+** | - | - |
| **59** | Tranebjærg et al. (1995) | M | Frameshift | Linkage / PCR | **+** | **+** | - | **+** | **+** | **+** | - | - | **+** | - | **+** | **+** | **+** | - | - |
| **60** | Tranebjærg et al. (1995) | M | Frameshift | Linkage / PCR | **+** | **+** | **+** | **+** | **+** | **+** | - | **+** | **+** | - | **+** | **+** | **+** | - | - |
| **61** | Tranebjærg et al. (1995) | M | Frameshift | Linkage / PCR | **+** | **+** | **+** | **+** | **+** | **+** | - | - | **+** | - | **+** | **+** | **+** | - | - |
| **62** | Tranebjærg et al. (1995) | M | Frameshift | Linkage / PCR | **+** | **+** | - | **+** | **+** | **+** | - | - | **+** | - | **+** | **+** | **+** | - | - |
| **63** | Tranebjærg et al. (1995) | M | Frameshift | Linkage / PCR | **+** | **+** | - | **+** | **+** | **+** | - | - | **+** | - | **+** | **+** | **+** | - | - |

| **ID Source** | **Sex** | **Mutation Type** | **Methods Used** | **SSP** | **PCB** | **D** | **E** | **DMP** | **A** | **MICM** | **FO** | **DM** | **CA** | **IR** | **DGB** | **DNO** | **DLD** | **XLA** |
| --- | --- | --- | --- | --- | --- | --- | --- | --- | --- | --- | --- | --- | --- | --- | --- | --- | --- | --- |
| **64** Tranebjærg et al. (2000) | M | Frameshift | Linkage / PCR | **+** | - | **+** | - | **+** | **+** | - | **+** | - | - | **+** | - | - | **+** | - |
| **65** Tranebjærg et al. (2001) | M | Frameshift | Linkage / PCR | **+** | **+** | - | - | **+** | **+** | - | - | - | **+** | - | - | **+** | - | - |
| **66** Tranebjærg et al. (2001) | M | Frameshift | Linkage / PCR | **+** | **+** | - | - | **+** | **+** | - | - | - | **+** | - | - | **+** | - | - |
| **67** Tranebjærg et al. (2001) | M | Frameshift | Linkage / PCR | **+** | **+** | - | - | **+** | **+** | - | - | - | **+** | - | - | **+** | - | - |
| **68** Ujike et al. (2001) | M | Point mutation | RFLP / Sanger | **+** | - | **+** | - | **+** | - | - | - | - | - | - | - | - | - | - |
| **69** Ujike et al. (2001) | M | Point mutation | RFLP / Sanger | **+** | - | **+** | - | **+** | - | - | - | - | - | **+** | - | - | - | - |
| **70** Ujike et al. (2001) | M | Point mutation | RFLP / Sanger | **+** | - | **+** | - | - | - | - | - | - | - | **+** | - | - | - | - |
| **71** Ujike et al. (2001) | M | Point mutation | RFLP / Sanger | **+** | - | **+** | - | - | - | - | - | - | - | - | - | - | - | - |
| **72** Ujike et al. (2001) | M | Point mutation | RFLP / Sanger | **+** | - | - | - | - | - | - | - | - | - | - | - | - | - | - |
| **73** Wang et al. (2019) | M | Frameshift | NGS | **+** | - | - | - | - | - | - | - | - | - | - | - | - | - | - |
| **74** Wang et al. (2019) | M | Frameshift | NGS | **+** | - | - | - | - | - | - | - | - | - | - | - | - | - | - |
| **75** Yu et al. (2020) | M | Frameshift | Targeted Seq. | **+** | - | - | - | - | - | - | - | - | - | - | - | - | - | - |
|  |  |  |  |  |  |  |  |  |  |  |  |  |  |  |  |  |  |  |

- **Legend:** MTS, Mohr-Tranebjærg syndrome; XLA, X-linked agammaglobulinemia; array CGH, array comparative genomic hybridization; FISH, fluorescence in situ hybridization; qPCR, quantitative polymerase chain reaction; NGS, next-generation sequencing; WES, whole exome sequencing; WB, Western blot; RFLP, restriction fragment length polymorphism. SSP = Progressive sensorineural deafness, PCB = Progressive cortical blindness, D = Dystonia, E = Spasticity, DMP = Progressive mental deterioration, A = Ataxia, MICM = Involuntary movements and muscle contractures, FO = Bone fractures, DM = Muscular dystrophy, CA = Cerebellar Ataxia IR = Reduced intelligence, DGB = Basal ganglia degeneration, DNO = Optic nerve degeneration, DLD = Delayed language development, ADS = Sexual development disorders, XLA = Immunodeficiency with agammaglobulinemia.
- **Notes:** Phenotypic presence marked with (**+**) indicates clinical presentation; (-) indicates absence or not reported. All patients in this cohort are male (M).

| **Table S2. List of excluded articles** | | |
| --- | --- | --- |
| **Nº** | **Title** | **Ref.** |
| 1 | Human deafness dystonia syndrome is a mitochondrial disease | (Koehler et al., 1999) |
| 2 | The mitochondrial TIM22 preprotein translocase is highly conserved throughout the eukaryotic kingdom | (Bauer et al., 1999) |
| 3 | Cloning and Expression of Mouse Deafness Dystonia Peptide 1 cDNA | (Nakane et al., 2000) |
| 4 | The role of the TIM8-13 complex in the import of Tim23 into mitochondria | (Paschen et al., 2000) |
| 5 | Role of the Deafness Dystonia Peptide 1 (DDP1) in Import of Human Tim23 into the Inner Membrane of Mitochondria | (Rothbauer et al., 2001) |
| 6 | Human deafness dystonia syndrome is caused by a defect in assembly of the DDP1/TIMM8a–TIMM13 complex | (Roesch et al., 2002) |
| 7 | The role of the Tim8p–Tim13p complex in a conserved import pathway for mitochondrial polytopic inner membrane proteins | (Curran et al., 2002) |
| 8 | Interaction of the deafness–dystonia protein DDP/TIMM8a with the signal transduction adaptor molecule STAM1 | (Blackstone et al., 2003) |
| 9 | The calcium-binding aspartate/glutamate carriers, citrin and aralar1, are new substrates for the DDP1/TIMM8a–TIMM13 complex | (Roesch et al., 2004) |
| 10 | Conformation-Sensitive Gel Electrophoresis as an Ideal High-Throughput Strategy for Accurate Detection of Sequence Variations in DNA: Screening hTomm and hTimm Genes | (Blesa et al., 2004) |
| 11 | Bax/Bak-Dependent Release of DDP/TIMM8a Promotes Drp1-Mediated Mitochondrial Fission and Mitoptosis during Programmed Cell Death | (Arnoult et al., 2005) |
| 12 | Differential Cerebral Cortex Transcriptomes of Baboon Neonates Consuming Moderate and High Docosahexaenoic Acid Formulas | (Kothapalli et al., 2007) |
| 13 | Retinal ganglion cell neurodegeneration in mitochondrial inherited disorders | (Carelli et al., 2009) |
| 14 | Neurological phenotype and reduced lifespan in heterozygous Tim23 knockout mice, the first mouse model of defective mitochondrial import | (Ahting et al., 2009) |
| 15 | A Distinct MicroRNA Signature for Defi nitive Endoderm Derived From Human Embryonic Stem Cells | (Hinton et al., 2010) |
| 16 | Alterations in expression levels of deafness dystonia protein 1 affect mitochondrial morphology | (Engl et al., 2012) |
| 17 | La vulnerabilidad de las células ganglionares de la retina a los defectos mitocondriales | (Solano Palacios, 2012) |
| 18 | Mutations in the phospholipid remodeling gene SERAC1 impair mitochondrial function and intracelular cholesterol trafficking and cause dystonia and deafness | (Wortmann et al., 2012) |
| 19 | Nuclear factors: Roles related to mitochondrial deafness | (Luo et al., 2013) |
| 20 | Function of hTim8a in complex IV assembly in neuronal cells provides insight into pathomechanism underlying Mohr-Tranebjærg syndrome | (Kang et al., 2019) |
| 21 | TIMM8A is associated with dysfunction of immune cell in BRCA and UCEC for predicting anti‑PD‑L1 therapy efficacy | (Zhu et al., 2022) |
| 22 | ncRNAs‑mediated high expresión of TIMM8A correlates with poor prognosis and act as an oncogene in breast cancer | (Wang et al., 2022) |
| 23 | Upregulation of TIMM8A is correlated with prognosis and immune regulation in BC | (Zhang et al., 2022) |
| 24 | Reduced mitochondrial size in hippocampus and psychiatric behavioral changes in the mutant mice with homologous mutation of Timm8a1-I23fs49X | (Ouattara et al., 2022) |
| 25 | Chicken GLUT4 function via enhancing mitochondrial oxidative phosphorylation and inhibiting ribosome pathway in skeletal muscle satellite cells | (Zhang et al., 2024) |
| 26 | TIM8 Deficiency in Yeast Induces Endoplasmic Reticulum Stress and Shortens the Chronological Lifespan | (Tang et al., 2025) |
| 27 | Identification of MIM23, a putative component of the protein import machinery of the mitochondrial inner membrane. | (Dekker et al., 1993) |
| 28 | Deafness-Dystonia-Optic Neuronopathy Syndrome. | (Tranebjærg, L., 2003) |
| 29 | Mitochondrial Respiratory Chain Complex II. | (Ganesh et al., 2013) |
